# Interplay of prefrontal cortex and amygdala during extinction of drug seeking

**DOI:** 10.1007/s00429-017-1533-9

**Published:** 2017-10-28

**Authors:** Valeria Oliva, Emilio Cartoni, Emanuele Claudio Latagliata, Stefano Puglisi-Allegra, Gianluca Baldassarre

**Affiliations:** 1 Department of Biology and Biotechnology Charles Darwin, University of Rome La Sapienza, Rome, Italy; 2 Laboratory of Computational Embodied Neuroscience, Institute of Cognitive Sciences and Technologies, National Research Council, Rome, Italy; 3 School of Physiology, Pharmacology and Neuroscience, University of Bristol, Bristol, UK; 4 Santa Lucia Foundation, IRCCS, Rome, Italy

**Keywords:** Noradrenaline, Extinction, Conditioned place preference, Medial prefrontal cortex, Amygdala, Leaky neurons

## Abstract

Extinction of Pavlovian conditioning is a complex process that involves brain regions such as the medial prefrontal cortex (mPFC), the amygdala and the locus coeruleus. In particular, noradrenaline (NA) coming from the locus coeruleus has been recently shown to play a different role in two subregions of the mPFC, the prelimbic (PL) and the infralimbic (IL) regions. How these regions interact in conditioning and subsequent extinction is an open issue. We studied these processes using two approaches: computational modelling and NA manipulation in a conditioned place preference paradigm (CPP) in mice. In the computational model, NA in PL and IL causes inputs arriving to these regions to be amplified, thus allowing them to modulate learning processes in amygdala. The model reproduces results from studies involving depletion of NA from PL, IL, or both in CPP. In addition, we simulated new experiments of NA manipulations in mPFC, making predictions on the possible results. We searched the parameters of the model and tested the robustness of the predictions by performing a sensitivity analysis. We also present an empirical experiment where, in accord with the model, a double depletion of NA from both PL and IL in CPP with amphetamine impairs extinction. Overall the proposed model, supported by anatomical, physiological, and behavioural data, explains the differential role of NA in PL and IL and opens up the possibility to understand extinction mechanisms more in depth and hence to aid the development of treatments for disorders such as addiction.

## Introduction

In the last decade, extinction of Pavlovian conditioning has gained interest as a possible means to treat disorders such as anxiety disorders, addiction, and eating disorders (Delamater and Westbrook 2014). Extinction is modelled in preclinical studies by non-reinforced exposure to previously conditioned stimuli in behavioural paradigms (Bernardi and Lattal 2010; McNally 2014). For example, in a conditioned place preference paradigm (CPP), a mouse learns to associate a drug with a particular environment, thus exhibiting a preference for such environment with respect to one not associated with the drug. Subsequent exposures to the same environment without the presence of the drug cause the extinction of the association measured as same preference for the two environments.

Neural substrates of extinction are still elusive but there is increasing evidence that brain regions such as the medial prefrontal cortex (mPFC), amygdala (Amg) and locus coeruleus (LC) play a crucial role in this process (Dunsmoor et al. 2015). In particular, noradrenaline (NA) from LC was recently shown to play a differential role in two subregions of the mPFC. NA causes an early extinction when depleted from the prelimbic (PL) region but impairs extinction when depleted from the infralimbic (IL) region in a CPP paradigm using amphetamine (Latagliata et al. 2016). What is still unclear is how NA in the mPFC can affect subcortical regions such as the Amg during Pavlovian conditioning and extinction.

In this research we analyse extinction through empirical experiments and with computational models. Although most existing models of extinction focus on fear-conditioning paradigms (Carrere and Alexandre 2015; John et al. 2013; Pendyam et al. 2013; Mannella et al. 2008; Moren and Balkenius 2000), they explore the dynamics of the Amg and of other structures involved in appetitive conditioning as well (Peters et al. 2009). For example, a model by Carrere and Alexandre (2015) shows how two different populations of the BLA can trigger either a conditioned response or its extinction thanks to cholinergic modulation. On the other hand, John et al. (2013) presented a model that focuses on the dynamics of the intercalated cell mass in both extinction and reinstatement. Only one computational model (Pendyam et al. 2013) investigates NA and proposes that this neuromodulator enhances the activity of PL during the conditioning phase of a fear-conditioning paradigm. However, to our knowledge no model defines the role of NA during extinction learning and explores the differential role of NA in PL and IL.

This paper presents a computational model that reconciles previous models with the latest experimental findings and proposes an explanation of various empirical results. The model reproduces the two experiments mentioned above, performed by Latagliata et al. (2016), where NA was depleted from PL or IL in a CPP paradigm. In addition, it successfully reproduces preliminary empirical results coming from our laboratory where mice were injected with prazosin in PL on the first day of extinction, and a new double depletion empirical experiment that we present in this paper. In this experiment we removed NA *contextually from both regions* of the mPFC during the extinction training. Since depletion of NA from PL and from IL had opposite behavioural results, it was indeed interesting to analyse the effect of a double depletion to further understand NA role in the two regions.

In addition we simulated two possible experiments, which serve as testable predictions. In one of these simulated experiment we removed NA from IL during only the first day of the extinction phase. In the second simulated experiment we increased NA in PL cortex during the first day of extinction.

We performed a sensitivity analysis on all parameters of the model, for example the learning rate that defines how quickly a particular connection changes, to understand how the behaviour of the model changes in relation to the values of parameters. Thanks to this additional analysis we evaluated the strength of the model predictions and we can propose possible experiments based on robust results.

The paper is organised as follows. We first describe the architecture of the model, then explain its functioning, and then show the results of the simulations. Next, we present the results of the empirical experiment. Finally, we discuss all results. We report in Appendix the methods of the experiment and technical/mathematical details of the model.

## Biology and model architecture

### Hypothesis

The mPFC is a critical region for both the acquisition and the extinction of drug-seeking behaviour (Peters et al. 2009). In particular, it seems that two different subregions of the mPFC are involved in different phases of learning: the activity of PL is established to be crucial for the acquiring and maintenance of drug seeking (Di Pietro et al. 2006; Erb et al. 2000; Laughlin et al. 2011), whereas the activity of IL is necessary for the extinction process (Peters et al. 2008).

A number of findings show that the role of NA in PL and IL mirrors the role of those two structures. Depletion of NA from the mPFC (especially PL) impairs the acquisition of CPP or conditioned place aversion (CPA) (Ventura et al. 2003, 2005, 2008), stimulation of $\beta$-adrenergic receptors in PL facilitates the retrieval of cocaine-associated memories (Otis et al. 2013). Instead, the depletion of NA from PL during the extinction phase accelerates the extinction process (Latagliata et al. 2016), depletion of NA from IL during the extinction phase completely impairs the extinction (Latagliata et al. 2016) and activation of $\beta$-adrenergic receptors in IL accelerates the extinction (LaLumiere et al. 2010).

Inactivation studies (Do-Monte et al. 2015; Sierra-Mercado et al. 2011) show that the activity of IL is only crucial for learning during extinction, but it is not necessary for the expression of conditioned behaviour. These findings are also consistent with another finding on NA: the activity of LC is shown to be very high only during the first phase of extinction (Bouret and Sara 2004). This suggests that NA is very important for learning during extinction, similar to how it is important for the conditioning of highly motivational salient stimuli (Ventura et al. 2008), but it may not be necessary for the expression of extinction and conditioned behaviour.

This statement leads to another important question: what region is actually needed for the expression of extinction and conditioned behaviour? How does it interact with the mPFC? A strong candidate for this role is the Amg, a crucial region for Pavlovian conditioning (LeDoux 2007; Mirolli et al. 2010). A subregion of the Amg, the central amygdala nucleus (CEA), is indeed known to be able to trigger drug seeking (Everitt et al. 2003) and the intercalated cell mass (ITC), a small GABAergic nucleus in the Amg, seems to play a crucial role in the extinction of drug seeking through its GABAergic projections toward CEA (Likhtik et al. 2008). ITC can be divided into a dorsal (ITCd) and a ventral (ITCv) subregion (Pare and Duvarci 2012) and receives substantial glutamatergic projections from the mPFC (Vertes 2004). In addition, ITC shows glutamate-dependent plasticity, and is a strong candidate as a site of learning for extinction memory (Royer and Pare 2002). In addition, a study using fear conditioning shows that the basal amygdala (BA) can be subdivided into two populations, one active when the animal is exposed to a CS (BAf) and the other when the animal is exposed to an extinguished CS (BAe) (Herry et al. 2008). We propose that ITC and BA work together in an interconnected network to trigger a conditioned behaviour or prevent its performance after extinction.

NA in the mPFC, acting on both $\alpha$-1 and $\beta$ adrenergic receptors, has an excitatory effect. In particular, it was shown that NA can cause facilitation of glutamate-evoked discharge when acting on $\alpha$-1 receptors (Devilbiss and Waterhouse 2000) and enhance the excitability of the mPFC to external glutamatergic inputs (Luo et al. 2014). In addition, it was recently shown that NA acting on both $\alpha$ and $\beta$ receptors triggers LTP (Maity et al. 2015).

In our model, the mPFC triggers plasticity events inside the Amg thanks to its projection to the basolateral amygdala (BLA) and ITC. Whether this process leads to conditioning or to extinction depends on which region of the mPFC is involved. If a glutamatergic input (in particular an input concerning the presence of an unconditioned stimulus—US) arrives to PL, it causes conditioning. Instead, if a glutamatergic input (i.e. an input concerning the absence of the US) arrives to IL it causes extinction. After conditioning or extinction, the activity of the LC and the mPFC is no more necessary to express or prevent behaviour because the Amg has already learned.

It is worth noting that most of the data on PL and IL cited here come from rodent works. The PL/IL distinction is not present as clearly in primates. Indeed, both afferent and efferent projections suggest that PL might be functionally homologous to the dorsolateral prefrontal cortex and IL to the orbitomedial cortex of primates (Vertes 2004). In addition, the structure of intercalated masses also presents some differences in primates: they are not organised into clusters but form a continuous net that is extended throughout the antero-posterior axis of the amygdala (Zikopoulos et al. 2016). Moreover, their inner circuit and function has yet to be clarified.

### Architecture of the model

**Fig. 1:**
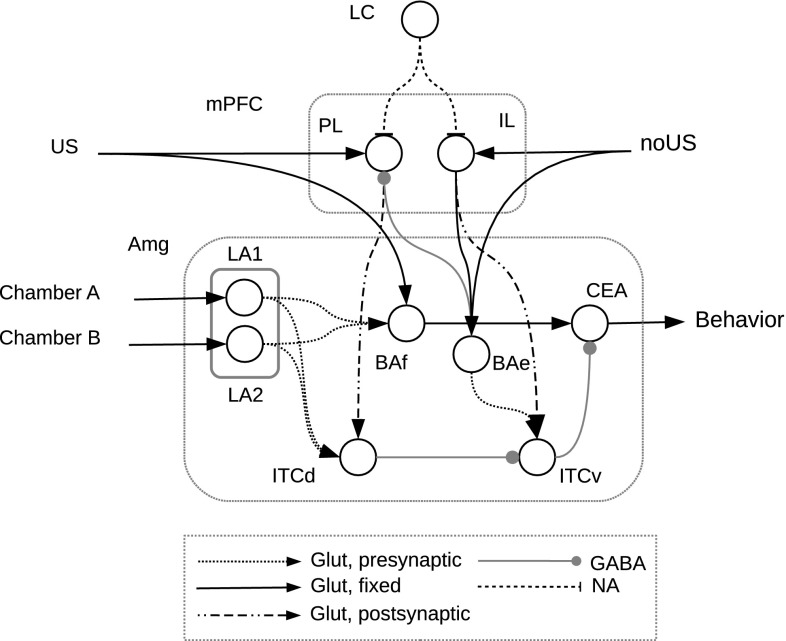
The model architecture is formed by these components: the locus coeruleus (LC), the medial prefrontal cortex (mPFC), which comprises the infralimbic (IL) and the prelimbic (PL) regions, and the amygdala (Amg), which comprises the lateral amygdala (LA), divided into two subpopulations, the basal amygdala divided into two subpopulations (BAe and BAf), the central amygdala (CEA), the dorsal intercalated cell mass (ITCd), and the ventral intercalated cell mass (ITCv). The model receives four different external inputs: the occurrence of US (US), the non-occurrence of US (noUS) and two stimuli (Chamber A, Chamber B) representing the two chambers of the CPP apparatus. Connections between structures are noradrenergic (NA), glutamatergic (Glut) or GABAergic (GABA). Glutamatergic connections can be either plastic (Glut, learning) or fixed (Glut)

The architecture of the model is shown in Fig. 1. It is formed by integrate-and-fire neurons to represent neural populations of the Amg and of the mPFC. The Amg is composed of four different regions: lateral amygdala (LA), basal amygdala (BA), ITC and CEA. We consider activity in CEA as the output of our model. We further divided BA into two different populations, BAf and BAe, and ITC in ITCv and ITCd, in accordance with experimental findings (Herry et al. 2008; Pare and Duvarci 2012). We also split LA into two populations, each receiving as input an activation representing a different chamber of the simulated CPP experiment. mPFC is represented by two different regions, PL and IL. External inputs signal the presence of chamber A or chamber B and the occurrence of the US or its non-occurrence (no US). BAf and PL receive an input representing the US, whereas BAe and IL cortex receive as input the no US. These inputs are abstract representations of external signals that arrive from multiple regions. For example, stimuli like the chambers are probably signalled by sensory cortices and the hippocampus, while the US might be signalled by the thalamus and somatosensory cortices (LeDoux 2007). The no US represents an expectation-violation and is produced when the US is missing after the animal had previously learned to expect it (this is not explicitly simulated in the model). The brain regions involved in this process might be the medial frontal cortex for the reward expectation and the anterior cingulate cortex (ACC) for the detection of the erroneous prediction (Alexander and Brown 2011; Silvetti et al. 2011, 2014)

The model also simulates the neuromodulator NA injected by LC to both regions of the mPFC. In the model, NA has an excitatory effect on mPFC populations and also amplifies incoming glutamatergic inputs (Luo et al. 2014; Devilbiss and Waterhouse 2000).

The connections forming the model architecture are grounded in the existing literature and are reported in Table 1. PL has glutamatergic projections to ITCd while IL projects glutamate to BAe and ITCv (Vertes 2004). LA has a glutamatergic projection to both BAf and ITCd, and BAf in turn projects to CEA. BAe projects to ITCv and to GABAergic interneurons in PL. ITCd has a GABAergic projection to ITCv, which, in turn, inhibits CEA through a GABAergic projection (Pare and Duvarci 2012).

In the model, the production of NA was not simulated and NA was rather given to the model as input. Indeed, the production of NA by LC involves complex processes, for example the detection of novel or unexpected or rewarding events (Sara 2009), that go beyond the focus of this work centred on NA effects on target structures.

Plasticity is present in the connections from PL to ITCd, from IL to ITCv, from LA to ITCd, from BAe to ITCv and from LA to BAf. Connections can in particular be either strengthened or weakened based on Hebbian learning. In particular, we use postsynaptic gated Hebbian learning (Gerstner and Kistler 2002) for PL–ITCd and IL–ITCv and presynaptic gated Hebbian learning (Gerstner and Kistler 2002) for LA-ITCd, BAe–ITCv and LA–BAf. This reflects the hypothesis that the connections from PL to ITCd and from IL to ITCv become stronger when the mPFC has an high firing rate, a condition that happens in particular when it is reached by high levels of NA. On the other hand, connections from LA to ITCd and from BAe to ITCv are hypothesised to be strengthened when the ITC neurons are very active: also this condition is initially caused by a high firing rate in mPFC. These mechanisms implement the overall idea of the existence of an important prefrontal control on the Amg plasticity processes. Connections from LA to BAf are strengthened when both areas are strongly active, and this happens only with the contemporary experience of the CS and US. The connections so formed represent the core CS–US association of Pavlovian learning (Mirolli et al. 2010).

**Table 1:** Bibliographical references supporting the existence of the connections forming the architecture of the model

Connection	References
PL to ITCd	Vertes (2004)
IL to ITCv	Vertes (2004)
LA to BAf	Pare and Duvarci (2012)
LA to ITCd	Pare and Duvarci (2012)
BAe to ITCv	Amano et al. (2010)
BAf to CEA	Pare and Duvarci (2012)
ITCd to ITCv	Pare and Duvarci (2012)
ITCv to CEA	Pare and Duvarci (2012)
BA to GABA in PL	Gabbott et al. (2006)
IL to BAe	Vertes (2004)

## Simulations and results

In each simulation we used the same CPP protocol used in the behavioural experiment (see “Materials and Methods” for details), with inputs given as described in Table 2 and shown in Fig. 3.

The results we now illustrate were obtained consistently by setting the model parameters to anyone of the parameter sets found by the sensitivity analysis illustrated below in Sect. 3.1.

**Table 2:** Simulated CPP protocol used to test the model. Some trials are repeated twice with presentation of chamber A and B (see the main text)

Phase	Number of trials	Inputs
Conditioning training	4	Chamber A + US + NA
Conditioning training	4	Chamber B
Conditioning test	1	Chamber A + noUS + NA
Conditioning test	1	Chamber B
Extinction training	14	Chamber A + noUS + NA
Extinction training	14	Chamber B
Reinstatement	1	US + NA
Reinstatement test	1	Chamber A + noUS + NA
Reinstatement test	1	Chamber B

**Fig. 2:**
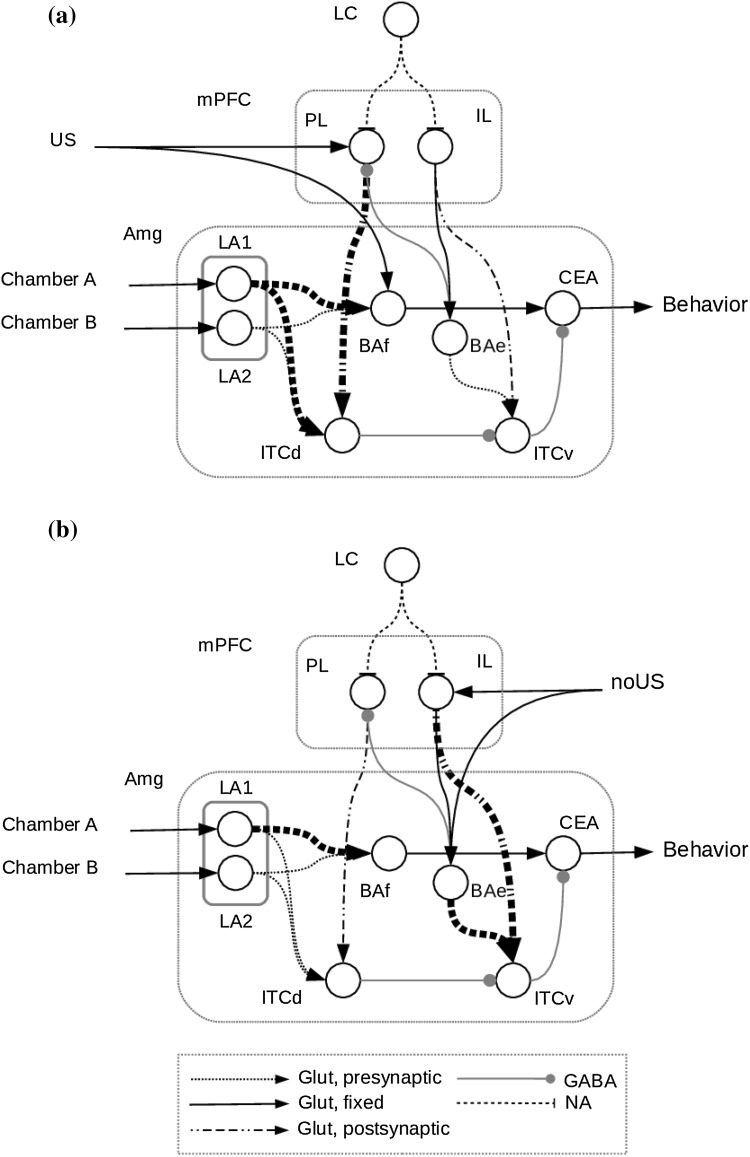
Strength of the model connection weights after **a** conditioning and **b** extinction. Thicker lines represent strengthened connections

During conditioning trials the model was exposed to chamber A with the US and NA, and then to chamber B. During this phase, connections from LA to BAf, from LA to ITCd and from PL to ITCd strengthen rapidly. In the meanwhile, connections from BAe and IL to ITCv remain weak (Figs. 2a, 4). This leads to an increase of CeA activity when the CS is present, therefore, triggering the conditioned response.

During the test phase we removed the US and added the noUS input. We exposed the model first to the CS (chamber A) and then to the non-conditioned chamber (chamber B). This was done to model the animal looking at each chamber and evaluating the possibility of approaching/remaining within it. We calculated the activity of CEA for each chamber exposure and compared the two values to find which chamber the model would choose. The activity of CEA was much higher during the exposure to chamber A, indicating that the conditioning was acquired. The connection from LA to BAf remained stable. On the other hand, the connections from PL to ITCd and from LA to ITCd weaken as chamber A was presented without US during the test.

The extinction phase was identical to the test phase but NA was high for the first 3 days, and then slowly decreased arriving to 0 in day 8. Then extinction continued until day 14. This simulates the fact that NA gradually diminishes during extinction (Sara 2009). During this phase the connection from LA to BAf remained stable. On the other hand, connections from IL and from BAe to ITCv were strengthened (Figs. 2b, 4). As in the test phase, we evaluated the activity of CEA during each chamber exposure. We considered the conditioning extinguished when the activity of CEA was no longer high during the exposure to the conditioned chamber compared to the exposure to the other chamber.

We added a reinstatement phase directed to verify that the model did not completely forget the conditioning but reinstated the preference after just one presentation of the US, in consistency with behavioural studies (Tzschentke 2007).

Then we tested again the preference for one trial as described above: CEA activity showed again a preference for chamber A. This happens because the connection between LA and BAf remains strong during the extinction, hence a single presentation of the US is enough to let the conditioned behaviour to re-emerge.

**Fig. 3:**
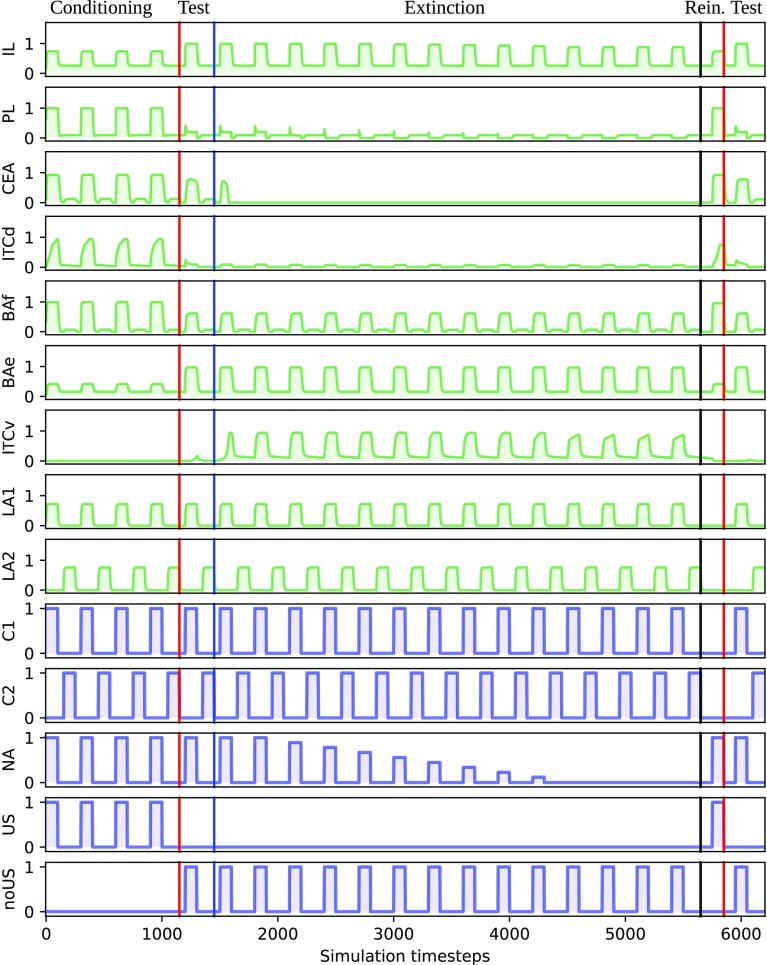
Example of inputs and the consequent activation of the model neuronal populations in a typical simulated control simulation. Red lines indicate the beginning of a test phase, the blue line indicates the beginning of the extinction phase and the black line indicates the beginning of the reinstatement phase. C1 is chamber A, C2 is chamber B, LA1 is the population of LA receiving C1 as input, LA2 is the population of LA receiving C2 as input

**Fig. 4:**
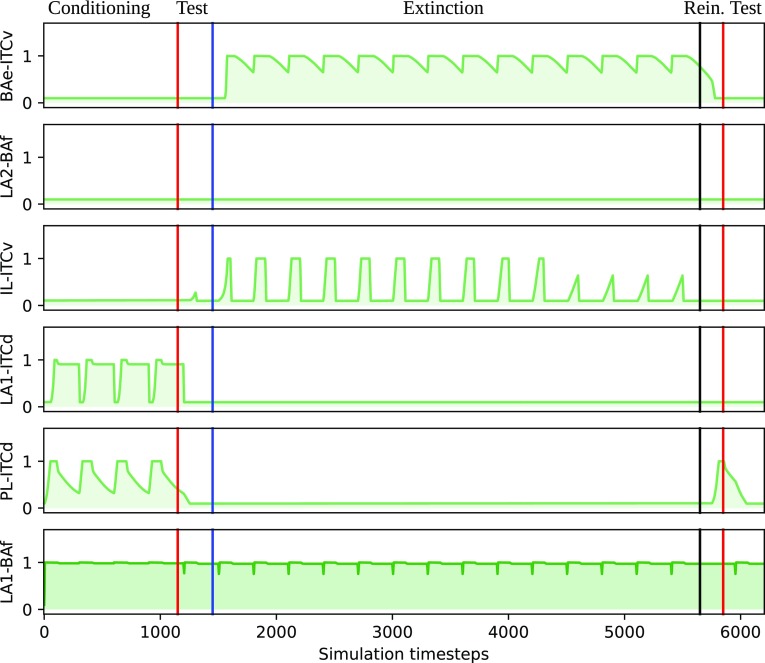
Weights dynamics during a typical simulation of the control condition. Red lines indicate the beginning of a test phase, the blue line indicates the beginning of the extinction phase and the black line indicates the beginning of the reinstatement phase

Figure 3 shows the temporal evolution of the inputs and of the activity of the model neuronal populations, while Fig. 4 shows synaptic weights during the whole simulation.

**Fig. 5:**
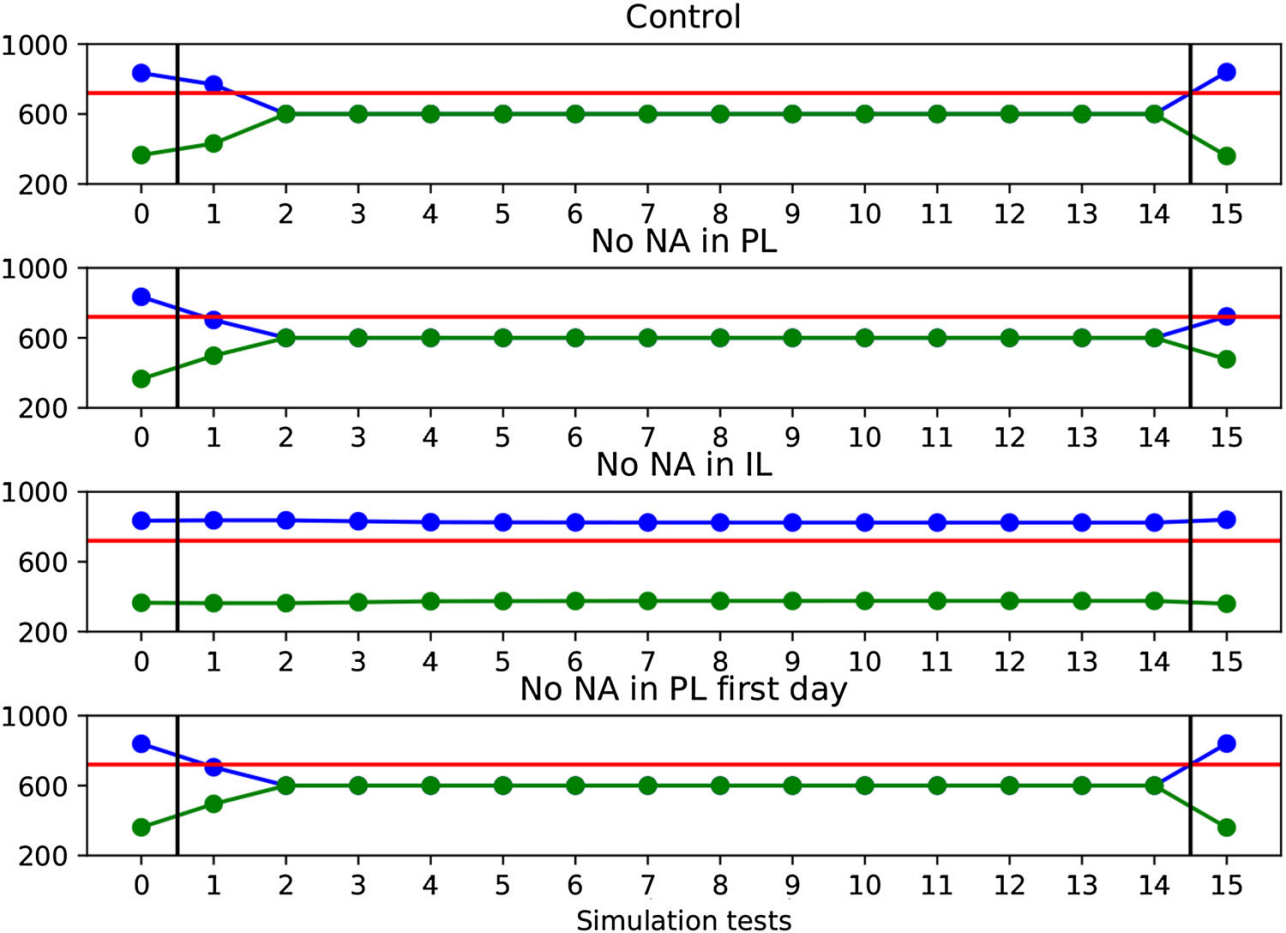
The figure shows how the model behaves during four simulated manipulations plus the control (no manipulation) in 1 day of conditioning test (day 0), 14 days of extinction (day 1–14), and a final restatement test (day 15). The blue curve indicates the preference for chamber A, while the green curve shows the preference for chamber B. The *y*-axis represents the time spent in each chamber. The red line represents a threshold below which we consider the behaviour extinguished. Note that for all the experiments shown we have empirical data only for conditioning and extinction. The presence or absence of reinstatement can, therefore, be considered another prediction of the model

Using this paradigm, we performed eight simulations in different conditions (seven experimental manipulations plus one control simulation, see Table 3). The first two simulations were based on Latagliata et al. (2016) experiments: in the first simulation we removed NA from PL during the extinction training, in the second simulation we removed NA from IL during the extinction training. The model without NA in PL region during the extinction phase extinguishes the preference already on the first day (Fig. 5). On the other hand, the model without NA in the IL region during the extinction phase does not extinguish the conditioned response after 14 trials. Both simulations mirror the results from Latagliata et al. (2016).

In a third simulation, NA was depleted from PL only on the first day of extinction, obtaining a similar result to the depletion of NA from PL during the whole extinction phase. Indeed, as shown in Fig. 5, the model extinguishes the preference earlier than the control group. This simulation was based on some preliminary results from our laboratory, in which an injection of prazosin in PL caused an early extinction (Latagliata 2014).

Next, we removed NA from both PL and IL during the extinction phase, similar to what done in the depletion experiment presented below in this paper. In this simulation, the model did not extinguish the acquired preference after 14 days: the probability of choice of chamber A remained high in every trial, as shown in Fig. 5.

**Table 3:** Experimental manipulations used in the sensitivity analysis. The first four experiments (first four rows) were used to search the parameters of the model, whereas the last two, related to the model predictions, were tested for their robustness with respect to the found model parameters. The fifth row refers to the result empirically tested here (also this result was obtained with the model parameters found with the sensitivity analysis)

Treatment	Result	References
NA depletion from PL during extinction training	Faster extinction	Latagliata et al. (2016)
NA depletion from IL during extinction phase	Extinction impaired	Latagliata et al. (2016)
$\alpha$-1 blockade of PL during the first day of extinction phase	Faster extinction	Preliminary results Latagliata (2014)
IL inactivation during the second part of extinction phase	Normal extinction	Do-Monte et al. (2015)
$\alpha$-1 blockade of IL during the first day of extinction phase	Extinction slowed	Prediction to be tested in a future experiment
Addition of NA in PL during the first day of extinction phase	Extinction slowed	Prediction to be tested in a future experiment
NA depletion from PL and IL	Extinction impaired	Verified with experiment presented in this paper

### Predictions and sensitivity analysis

We also performed two additional simulations to propose new experiments and predict possible results. First, we removed NA from IL only on the first day of extinction, to mirror the experiment with prazosin in PL. In the second simulation we increased NA input to PL during the extinction phase. In both simulations, the manipulation tended to slow extinction learning (results shown below).

We performed a sensitivity analysis on all parameters of the model both to find its parameters and to check the robustness of the predictions, i.e. to check if they held under different parameter values. To this purpose, we randomly sampled 98 millions randomly generated different combinations of parameter values and evaluated the behaviour of the model under all these settings. We ran all the simulations on the Neuroscience Gateway Portal (Sivagnanam et al. 2013).

The experiments involved in the sensitivity analysis are summarised in Table 3. We used results from previous experiments [i.e. NA depletion studies, Latagliata et al. (2016), and inactivation studies, Do-Monte et al. (2015)] as constraints to define the parameter values considered as acceptable. Thus, each combination of parameters had to replicate the behaviour of rats under the control condition and also replicate the results obtained under the experimental manipulations listed in Table 3 to be considered a plausible set of values (“valid sample”—see also “Materials and Methods”).

Out of 98 millions samples, 103 samples satisfied all the experimental constraints. The small number of these samples indicates that the model has been highly constrained with respect to its degrees of freedom, so its behaviour is reliable.

**Fig. 6:**
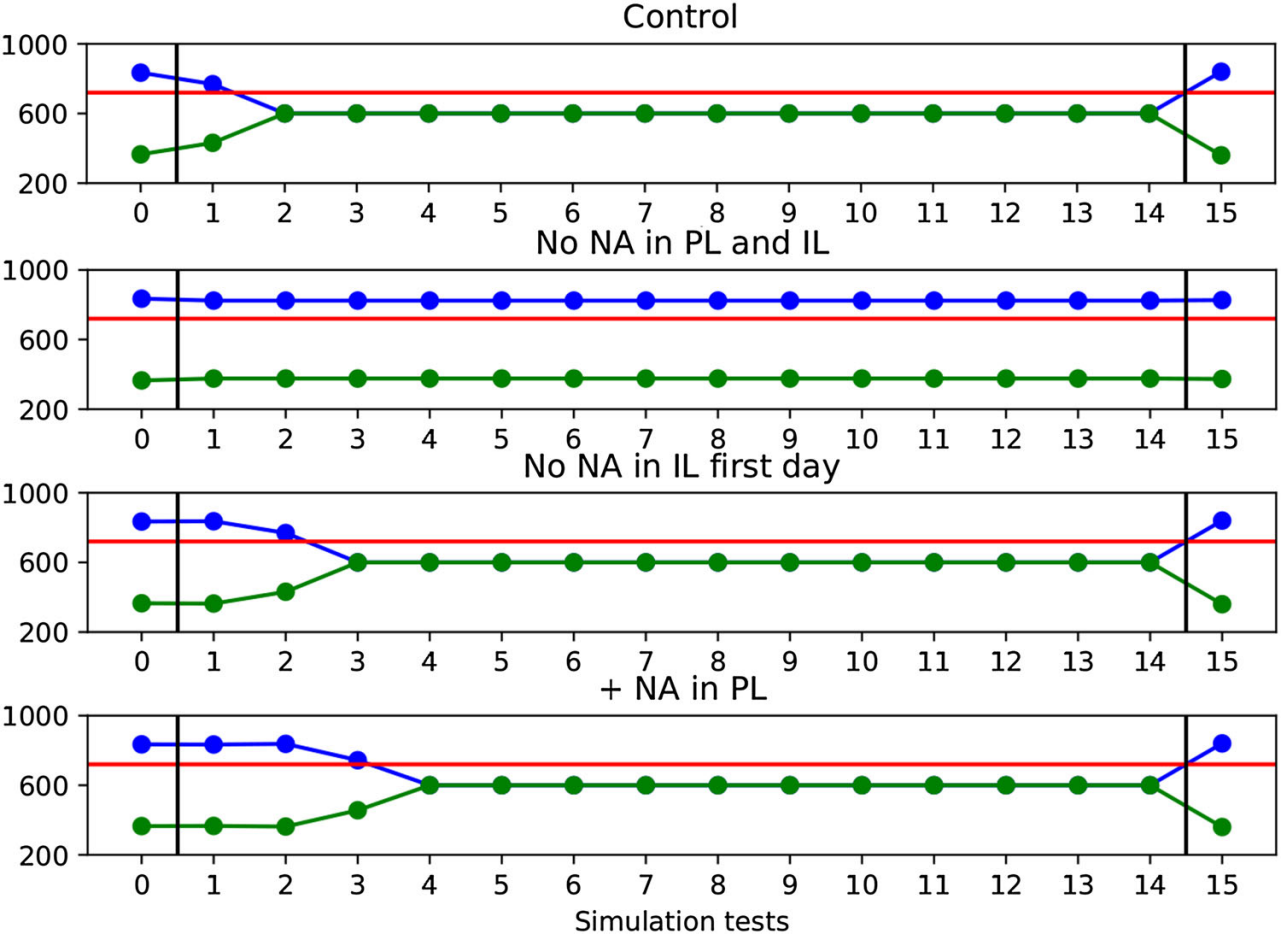
The figure shows an example of the model behaviour, with respect to the two predictions it produced, with a sample of parameters found by the sensitivity analysis. For both conditions related to the two predictions, extinction of the preference for one chamber is slowed compared to the control group. Axes are defined as in Fig. 5

In the simulation where NA was removed from PL and IL, 98 out of 103 never showed extinction (Fig. 6). Only 5 out of 103 samples extinguished the preference on the same day of the control.

In the simulations where NA was removed from IL only on the first day of extinction, 83 out of 103 samples showed a slower extinction than controls (Fig. 6). Only 20 out of 103 samples extinguished the preference on the same day of the control.

In the simulations where NA was added in PL on the first day of extinction, 96 samples extinguished slower than controls (Fig. 6). Only 7 samples extinguished the preference on the same day of the control.

In conclusion, the majority of samples that fit past experiments data exhibit a slower extinction than controls, both when adding NA in PL and when removing NA from IL during the first day of extinction. On the other hand, when removing NA from both PL and IL the majority of samples never show extinction. The other samples extinguish on the same day of controls, with no samples showing a faster extinction. The sensitivity analysis show that these predictions are robust given the empirical constraints used to find the model parameters and the empirical evidence used to build the model architecture and functioning.

The first prediction (NA depletion from PL and IL) was tested using 6-hydroxydopamine as discussed in the following section.

The second prediction ($\alpha$-1 blockade in IL on the first day of extinction slows extinction learning) could be tested in real animals using prazosin, thus mirroring the experiment where prazosin was injected in PL (Latagliata 2014), while the third prediction (the addition of NA in PL on the first day of extinction slows extinction learning) could be performed using an adrenergic agonist.

## Experiment: double NA depletion from PL and IL

### Behavioural paradigm

The apparatus used for the CPP consists of a three-compartment chamber with the outer compartments that have different characteristics. The experiment begins with a pretest habituation phase in which the mouse is free to explore the new environment. Later, during conditioning training, the animal is injected with a drug and is then placed into one of the compartments for several minutes. On the following day, the mouse is injected with the drug vehicle and then placed in the opposite chamber.

The experiment used 13 mice as subjects. In the test phase the animal is placed at the central compartment and is allowed to explore the entire apparatus. The time the animal spends in each compartment is measured and a CPP is considered found if the animal spends significantly more time in the drug-paired compartment (CS) versus the vehicle-paired compartment.

Animals that are subjected to surgery undergo a further test (re-test) to check whether surgery for NE depletion has affected the place preference. The day after the re-test, the extinction procedure begins.

The extinction phase is exactly like the test phase and lasts until the mouse has extinguished for at least two consecutive days. The CPP is considered extinguished when the mouse spends a similar amount of time in both chambers of the apparatus (Cunningham et al. 2006). See “Materials and Methods” for details.

### Results

**Fig. 7:**
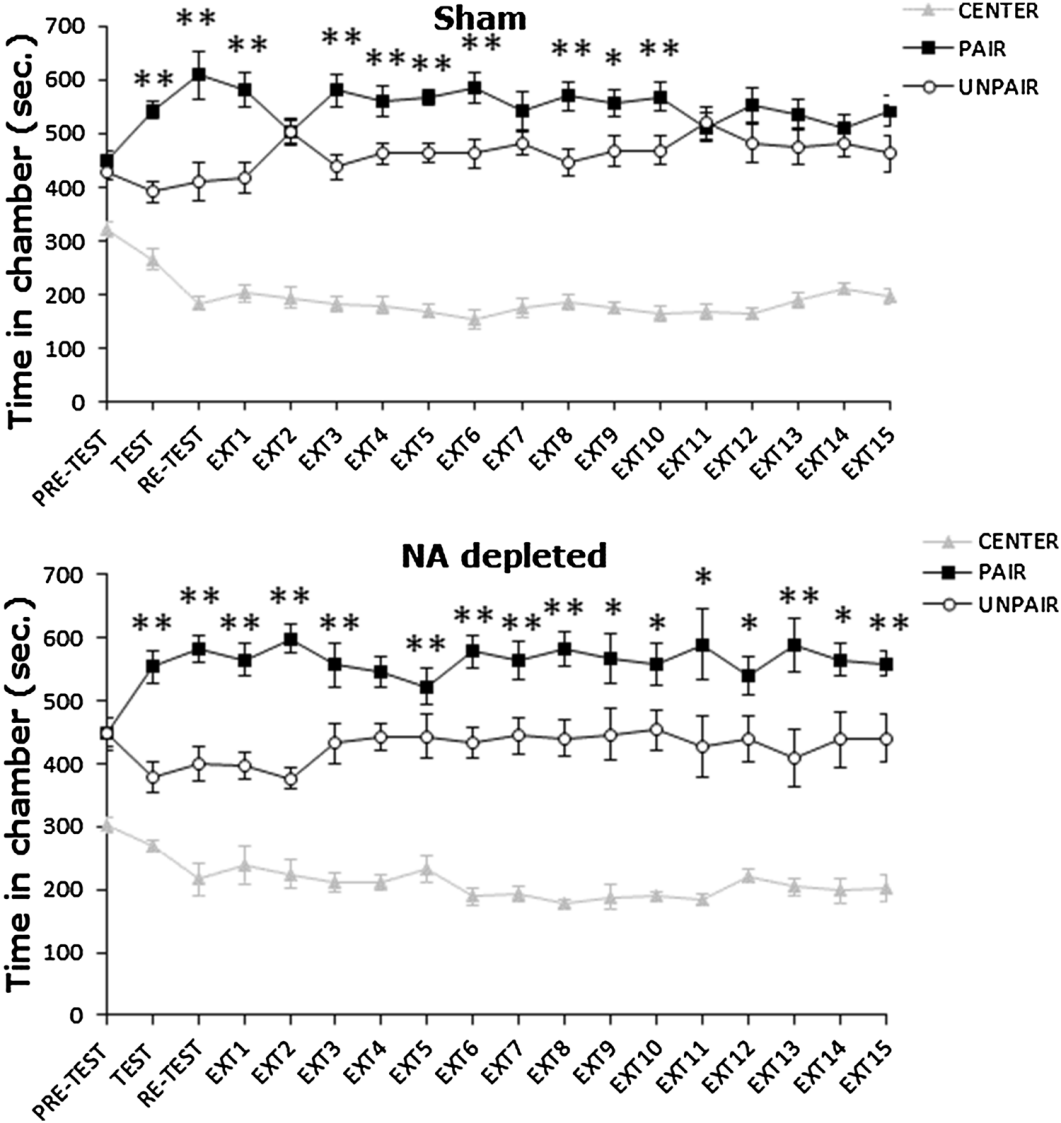
Effects of selective NA depletion in PL and IL prefrontal cortices on expression and daily extinction trials of an acquired conditioned place preference (CPP) induced by 2.5 mg/Kg of amphetamine. Time spent in the centre, paired and unpaired chamber during pretest, test, re-test and non-confined extinction trials in animals assigned to Sham and NA-depleted groups. All data are expressed as mean (sec.) time spent in center, paired and unpaired chambers. The symbols * and ** indicate the statistical significance of the preference for the paired chamber compared to the unpaired one (*p < 0.05, **p < 0.01)

During the pretest phase all mice, randomly assigned to Sham or NA-depleted groups, spent an equal amount of time (mean ± SEM) in the two lateral chambers, thus showing that the apparatus was unbiased in terms of preferences in untreated mice (see Fig. 7). A one-way ANOVA of the pretest revealed a significant effect of the factor choice for Sham [*F*(2,18) = 22.865, *p* < 0.01] and NA-depleted mice [*F*(2,15) = 15.629, *p *< 0.01]. Post hoc comparisons confirmed that mice spent more time in the lateral chambers than in the centre (centre vs paired *p* < 0.01, for all groups).

Following the conditioning phase all animals showed a preference for the chamber previously paired with amphetamine. An ANOVA on the CPP test results showed a significant main effect of the factor choice for Sham [*F*(2,18) = 56.044, *p* < 0.01] and NA-depleted mice [*F*(2,15) = 45.989, *p *< 0.01]. Duncan’s post hoc analyses confirmed that during the test both Sham (*p* < 0.01) and NA-depleted mice (*p* < 0.01) spent more time in the amphetamine-paired chamber in comparison with saline-paired chamber (Fig. 7).

NA depletion in mPFC cortex performed the day after CPP test did not affect the expression of amphetamine CPP on the re-test day. Indeed, a one-way ANOVA revealed a main effect of the factor choice for Sham [*F*(2,18) = 41.572, *p* < 0.01] and NA-depleted mice [*F*(2,15) = 55.067, *p *< 0.01]. Duncan’s post hoc test confirmed that both Sham (*p *< 0.01) and NA-depleted groups (*p* < 0.01) spent more time in the amphetamine-paired chamber than in the saline-paired chamber (Fig. 7).

As shown in Fig. 7, NA-depleted mice did not extinguish the preference for the conditioned chamber after 15 days (when the experiment was interrupted), while Sham animals extinguished on days 11–12. On extinction trials 11 and 12, respectively, statistical analyses revealed a significant main effect of the factor choice for Sham animals [having, respectively, *F*(2, 18) = 63.412, *p *< 0.01 and *F*(2, 18) = 51.707, *p* < 0.01 in the two conditions]. Duncan’s test showed that on both days Sham animals spent an equal amount of time in both lateral chambers (*p* = ns). On the other hand, NA-depleted mice spent on both days more time in the amphetamine than in the saline-paired chamber (*p *< 0.05). On day 15, a one-way ANOVA revealed a main effect for the factor choice for NA-depleted mice [*F*(2,15) = 58.600, *p *< 0.01]. Duncan’s post hoc test confirmed that NA-depleted mice *p *< 0.01 still spent more time in the amphetamine-paired chamber than in the saline-paired chamber (Fig. 7).

## Discussion

### Simulations, predictions and sensitivity analysis

Our model proposes that the behaviour observed in mPFC NA depletion experiments is caused by enhanced or impaired plasticity events triggered by mPFC in the Amg. In the model, connections from LA to ITCd and from BAe to ITCv have an opposite role on functioning and learning. In particular, LA–ITCd connections are strengthened during the conditioning phase and allow the conditioned response to be expressed thanks to the inhibition of ITCv. On the other hand, BAe-ITCv connections only grow during the extinction phase and allow the ITCv to inhibit the conditioned response triggered by CEA. Importantly, this effect is modulated by NA in the mPFC. Indeed, when NA reaches PL this region is activated, the connection from PL to ITCd is strengthened, and this causes the connection from LA to ITCd to learn as well. The exact same mechanism is proposed for the connections from IL to ITCv and from BAe to ITCv. As a consequence, depletion of NA from PL during extinction training causes an early extinction because the connection from PL to ITCd and from LA to ITCd starts to weaken earlier compared to the control group, an effect caused by PL firing at a lower rate without NA. As a consequence, both connections from IL to ITCv and from BAe to ITCv start strengthening earlier. The final effect is that CEA is inhibited earlier than the control group, therefore, leading to an anticipated extinction.

The opposite happens when NA is depleted from IL. Since IL without NA fires at a low rate, connections from IL to ITCv and from BAe to ITCv are never strengthened and CEA is never inhibited. A double depletion of NA from both regions causes the very same result. Again, connections to the ITCv are never strengthened and despite the connection from LA to ITCd being weakened, the connection from LA to BAf remains stable and this is enough to trigger a conditioned response. This result, confirmed by the behavioural experiment, shows that the effect of a NA depletion from IL cannot be reversed by a NA depletion from PL.

Furthermore, the model shows that if NA is depleted from PL only on the first day of extinction training, the extinction happens before the control, and connections inside the Amg behave on the overall as they do in the condition of complete depletion of NA from PL. This result is consistent with preliminary behavioural results from our laboratory where NA is depleted on the first day with an injection of prazosin in PL (Latagliata 2014).

The sensitivity analysis showed that with other weight dynamics the model can still reproduce all experiments. For example, in 37 out of 103 samples, the weight from LA to ITCd does not ever strengthen, while all other dynamics remain the same. When this happens the activity of ITCd is strictly dependent on the activity of PL: as soon as PL stops firing, ITCd stops inhibiting ITCv and allows the extinction. These samples, while reproducing all experiments correctly, do not capture the idea of the mPFC teaching the Amg to learn new CS–US associations, as stated in the hypothesis. Indeed, the connection from LA to ITCd is proposed by many authors (Pare and Duvarci 2012; John et al. 2013; Busti et al. 2011) to be important to trigger the activation of ITCd, but our finding suggests that PL to ITCd may be an alternative route. A further analysis, for example a disconnection of LA from ITCd, could clarify this issue.

Using the model we also proposed two possible novel experiments where, respectively, NA is removed from IL or NA is added in PL during the first day of extinction. The first experiment could be performed in real animals using prazosin, whereas the second could be performed using an adrenoreceptor agonist. The results, which hold for all parameter sets found by the sensitivity analysis, showed that in both cases the extinction is slowed in, respectively, 83 out of 103 samples and 96 out of 103 samples. In those samples, the model behaves as if the extinction training started 1 day after the control group. In the experiment where NA is removed from IL on the first day, the weights to ITCv start growing 1 day after the control group, while in the experiment were NA is added to PL the weights of connections to ITCd start weakening 1 day after the control group.

For setting the parameters and running the sensitivity analysis we used as constraints the experimental data from both appetitive and aversive conditioning studies. Indeed, neural substrates that underlie both processes seem to overlap, especially regarding the regions that we analysed (Peters et al. 2009). However, it should be noted that in fear-conditioning paradigms both the acquisition and extinction of Pavlovian conditioning are usually much quicker than in appetitive conditioning, likely due to the higher biological valence of survival in an aversive context. Notwithstanding these differences, we reproduced both paradigms using the same set of parameters because our idea is that, apart from the speed of learning, a manipulation in one of the regions of the model would have the same qualitative effect with both appetitive and aversive stimuli (see “Conclusions and future perspectives”).

### The role of PL and IL

As reviewed by Moorman et al. (2015) many studies in the literature show that PL and IL have distinct roles in drug seeking as well as in fear conditioning. Many findings show that PL triggers conditioned responses during both the conditioning phase (Burgos-Robles et al. 2009; Schroeder et al. 2001) and reinstatement (Capriles et al. 2003; Di Pietro et al. 2006) and identify two connections that might be especially important in these processes: the one from PL to BLA (Mashhoon et al. 2010) and the one from PL to the core of the nucleus accumbens (Ma et al. 2014). On the other hand, a number of findings implicate IL in the extinction of reward and fear-related behaviours (Peters et al. 2008; LaLumiere et al. 2012; Ovari and Leri 2008; Rhodes and Killcross 2004, 2007) thanks to its projections to ITC (Amano et al. 2010) and to the shell of the nucleus accumbens (Millan et al. 2011).

Based on all these findings, many authors have proposed a simple go/stop model for PL and IL, where PL is thought to be able to trigger a particular conditioned response and IL to cause the extinction of it (Gass and Chandler 2013; Ma et al. 2014; Peters et al. 2009; Van den Oever et al. 2010). However, such a model was criticised by Moorman et al. (2015) and considered an oversimplification because the mPFC is a complex region, involved in different functions and in different networks (Bissonette et al. 2013; Cassaday et al. 2014; Dalley et al. 2004; De Bruin et al. 2000; Kesner and Churchwell 2011; St Onge and Floresco 2010). For example, other studies report that PL inactivation fails to disrupt drug seeking (Weissenborn et al. 1997; Zavala et al. 2003) and present these results as evidence of the inaccuracy of the go/stop model.

Our model proposes a possible solution for those apparent inconsistencies. Indeed, within the model PL is not always necessary to acquire the conditioning while NA in PL is needed to acquire a conditioned response to high salience stimuli (Ventura et al. 2003, 2005, 2008, 2007). The activity of PL is definitely crucial for reinstatement (Capriles et al. 2003). In summary, when there is a complete inactivation of PL, the Amg acquires the CS–US association and is able to trigger a conditioned response. When NA is depleted from PL, this region has a low firing rate and causes the connections towards the ITCd to remain weak, leading to an impairment in the acquisition of the conditioning. On the other hand, when PL receives NA during the conditioning phase, it fires at an high firing rate, triggering plasticity events in the Amg. Those events will also be important in the reinstatement phase. A previous computational model on fear conditioning (Pendyam et al. 2013) proposed a similar hypothesis: in their model, supported by experimental findings, NA acting on $\beta$-receptors in PL is crucial for the acquisition of the conditioned response because it triggers a sustained activity in PL that is then able to influence the activity of BLA, leading to fear expression.

The role proposed for IL is similar but specular. NA in this region was shown to be crucial during the extinction phase (Bernardi and Lattal 2010; Latagliata et al. 2016). We propose that this neuromodulator is especially important during early extinction because it triggers synaptic plasticity events that allow the Amg to learn. Indeed, according to Mueller and Cahill (2010) and experimental evidence (Bouret and Sara 2004; Sara and Segal 1991), in late extinction NA release is no longer required as plasticity cascades have already taken place. In addition, IL activity is not necessary for the expression of behaviour, as shown by the findings of Do-Monte et al. (2015) and Sierra-Mercado et al. (2011) where an inactivation of IL after the extinction training does not impair extinction expression.

### The role of the amygdala

Regarding the Amg, we integrated ideas from different computational models to build a new architecture that is also constrained by physiological and anatomical findings. A main feature of the architecture is that its behaviour is controlled by the mPFC. In Carrere’s model (Carrere and Alexandre 2015) BA is split into two populations, a “fear population” and an “extinction population”, in accordance with the experimental finding by Herry et al. (2008). Indeed, both Carrere and Herry propose that extinction learning takes place in BA thanks to the extinction population. On the other hand, the model proposed by Moustafa et al. (2013) hypothesises that extinction takes place thanks to the direct connection from IL to ITC, leaving to BA a role only during the acquisition of the conditioned response. The model proposed here reconciles these two views thanks to a double projection from IL to both BA and ITCv. In agreement with a finding by Laurent et al. (2008), that reports that rats with BA lesions can indeed extinguish a fear response but do not show extinction if tested the day after, we propose BA as a site of retention of extinction and IL–ITCv connection as an alternative route that permits a temporary extinction in absence of BA. In line with this interpretation, ITCv is the ITC region that is crucial for extinction thanks to its inhibitory projection to CEA, and its activity can be triggered by IL as shown in Li et al. (2011). On the other hand, ITCd is active during the acquisition of conditioning memories, as shown in a study by Busti et al. (2011) where fear conditioning enhances expression of activity markers in this region, but not in ITCv. ITCd has an inhibitory projection to ITCv (Pare and Duvarci 2012) and is thought to be important for the reinstatement of the conditioned response as well (John et al. 2013). This double inhibition system allows to change how CEA responds to stimuli thanks to inputs coming from the mPFC. Note that in John et al. (2013) model learning rates in the ITC are hypothesised to be faster than those in the BL complex (LA and BA). This allows their model to quickly extinguish the Pavlovian conditioning without losing the CS–US associations and a rapid reinstatement when the US is present again. Although we initially used this idea in the model, the sensitivity analysis showed that in our model there is no need to hypothesise a difference in learning rates between the ITC and the BL complex. Indeed, in our model, regardless of the learning rates, the connection between LA and BA remains strong, with the flexibility of responding depending on ITC learning driven by mPFC as explained above. This happens because during the extinction LA is activated by the CS, it, therefore, activates BAf above threshold and causes the connection to remain strong (see presynaptic Hebbian rule, section 8.3). The conditioned behaviour is, however, not expressed thanks to the inhibition of CEA by ITCv.

## Conclusions and future perspectives

The present study sheds some light on the role of NA in the mPFC during extinction of drug seeking. This neuromodulator signals the presence of an high salience stimulus or the unexpected absence of it, and allows PL and IL to express their role. While both cortices receive NA at the same time and are both active during all phases of Pavlovian conditioning, they influence the Amg in opposite ways. PL is indeed dominant during the conditioning phase and causes plasticity events in the Amg. On the other hand, during the extinction phase IL prevails and drives the Amg to learn the new association CS-no US. When the US is present again, PL is dominant again and causes the Amg to quickly return to the expression of the conditioned response. In this context, NA is a main actor, being able to trigger plasticity events that are then expressed at the subcortical level.

Although the proposed model offers an explanation to many experimental results, there are still a number of open issues to address. First, it would be interesting to investigate how NA is itself triggered in early extinction and then decreased in late extinction. Exploring afferent connections to the LC and understanding how a prediction confirmation or a prediction error are signalled (Alexander and Brown 2011; Silvetti et al. 2011, 2014), can help to build a model where NA is autonomously regulated.

In addition, as stated above, neural substrates for extinction of appetitive and aversive tasks seem to be overlapping (Peters et al. 2009). Due to this overlapping, we decided to use, among the other constraints, the results of Do-Monte et al. (2015) which were obtained in a fear-conditioning paradigm. It would be interesting to perform the same optogenetic manipulations performed by Do-Monte et al. (2015) on mice undergoing a CPP paradigm, to confirm the validity of this hypothesis. Such studies would help to understand to what extent extinction circuits for fear and addiction overlap.

## Appendix A

### Computational details on the model

#### Integrated-and-fire neurons

The presented model was coded in Python programming language, using the packages NumPy, SciPy and Matplotlib (Jones et al. 2001; Perez et al. 2011; van der Walt et al. 2011; Hunter 2007). The system-level architecture of the model was built by following modelling principles developed in previous works to study the role of amygdala in conditioning (Mannella et al. 2010; Mannella and Baldassarre 2015; Mannella et al. 2016). With the purpose of simplifying the model and reducing the number of parameters, each region is modelled through a single computational unit representing a population of real neurons. The only exception is LA that is formed by two neural subpopulations, each receiving a different external stimulus as input.

The integration of the model equations was done through the Euler method with a timestep set to 0.001.

To represent each neuronal population we used a leaky unit, capturing the average firing rate of the related neural population, working as follows:

1 $$\begin{aligned} \tau \cdot \dot{u} = - u + I \end{aligned}$$

where *u* is the activation potential of the unit, $\dot{u}$ is the activation rate of change in time (derivative) of such potential, *I* is the input, and $\tau$ is a time constant regulating the overall speed of the unit dynamics, set to 0.005. The activation of the neural populations (i.e. their average firing rate) is based on a hyperbolic function of their activation potential:

2 $$\begin{aligned} v = [tanh(u)]^{+} \end{aligned}$$

where *v* is the unit activation, *tanh* is the hyperbolic tangent function and $[x]^{+}$ is a function returning 0 if *x* $\le$ 0 and *x* if *x* > 0.

Units not modulated by NA receive as input a vector comprising external inputs (i.e. US, noUS or CS) and the activation of afferent neurons. Each external input is encoded with a value of 0 or 1 depending on the fact that it is, respectively, absent or present. The overall input to each unit is as follows:

3 $$\begin{aligned} I = \sum _{i}[w_{i} \cdot v_{i}] \end{aligned}$$

where *I* is the sum of all inputs $v_{i}$ (both external inputs and inputs from other units of the model), each multiplied by the related connection weight $w_{i}$. Signals coming from GABAergic populations are represented through negative connection weights. Signals coming from external inputs are assumed to have a connection weight equal to 1.

#### Neuromodulation

To represent the effects of neuromodulation in the equations of the activation of neural populations, glutamate and NA inputs to the two cortical areas (PL and IL) are defined as follows (Fiore et al. 2015; Mannella et al. 2016):

4 $$\begin{aligned} I = (1 + w_{n} \cdot NA) \cdot [bl + (w_{v} \cdot v)] + (ad \cdot w_{n} \cdot NA) - (w_{b} \cdot BAe) \end{aligned}$$

where $w_{n}$ is the weight of the connection from LC, *NA* is the value of the noradrenaline input, *bl* is the baseline of the activation of the neural population (set to 0.2), $w_{v}$ is a connection weight, *v* is the value of the external input (US and noUS respectively for PL and IL), *ad* is a constant defining the size of the additive effect, $w_{b}$ is a connection weight and *BAe* is the input from basolateral amygdala (this latter inhibition term is present only for PL and is not affected by NA which is only reported to affect glutamatergic inputs (Luo et al. 2014)).

#### Learning rules

Weights from LA to ITCd, from BAe to ITCv, and from LA to BAf are updated according to the *presynaptic Hebbian rule* that makes the learning dependent on the activity of the presynaptic neuron (Gerstner and Kistler 2002). In the presynaptic rule if the presynaptic neuron is not active, the weight does not change. The activity of the postsynaptic neuron decides the direction of the weight change: if it is above the threshold the weight change will be positive, otherwise it will be negative.

Formally, the weight change is computed as follows:

5 $$\begin{aligned} \mathrm{d}W_\mathrm{ba} = \varepsilon _\mathrm{ba} (b - \theta _\mathrm{ba}) a \end{aligned}$$

where $dW_\mathrm{ba}$ is the change of the connection weight, $\varepsilon _\mathrm{ba}$ is the learning rate constant, *a* is the activation of the presynaptic neuron, *b* is the activation of the postsynaptic neuron, $\theta _\mathrm{ba}$ is the threshold above which the weight increases.

Weights from PL to ITCd and from IL to ITCv are updated according to the *postsynaptic Hebbian rule* that makes the learning dependent on the activity of the postsynaptic neuron (Gerstner and Kistler 2002). In the postsynaptic rule, if the postsynaptic neuron is not active the connection weight does not change. The activity of the presynaptic neuron decides the direction of the weight change.

Formally, the weight change is computed as follows:

6 $$\begin{aligned} \mathrm{d}W_\mathrm{ba} = \varepsilon _\mathrm{ba} b (a - \theta _\mathrm{ba}) \end{aligned}$$

All thresholds and parameters are shown in Table 4.

**Table 4:** Ranges of the parameters (minimum and maximum values) of the models that the sensitivity analysis found to satisfy all the required empirical constraints

Parameter	Meaning	Value
Fixed weights		
CS1–LA	Connection weight from CS to LA	0.4–2
CS2–LA	Connection weight from CS1 to LA	0.4–2
BAf–CEA	Connection weight from BAf to CEA	0.6–2
BAe–PL	Connection weight from BAe to PL	0.01–1.9
ITCd–ITCv	Connection weight from ITCd to ITCv	0.2–2
ITCv–CEA	Connection weight from ITCv to CEA	0.2–2
US–BAf	Connection weight from US to BAf	0.2–2
noUS–BAe	Connection weight from noUS to BAe	0.4–1.9
US–PL	Connection weight from US to PL	0.02–2
noUS–IL	Connection weight from noUS to IL	0.01–1.9
NA–IL	Connection weight from LC to IL	0.02–2
NA–PL	Connection weight from LC to PL	0.02–2
Initial values for learning weights		
LA–BAf	Connection weight from LA to BAf	0.01–0.2
LA1–BAf	Connection weight from LA1 to BAf	0.01–0.2
PL–ITCd	Connection weight from PL to ITCd	0.01–2
IL–ITCv	Connection weight from IL to ITCv	0.01–2
IL–BAe	Connection weight from IL to BAe	0.01–1
LA–ITCd	Connection weight from LA to ITCd	0.01–0.2
BAe–ITCv	Connection weight from BAe to ITCv	0.01–0.2
Parameters for plasticity		
*bl*	Baseline for PL and IL activation	0.1–0.6
*ad*	Size of additive effect of NA	0.01–2
$\theta _{LI}$	Threshold for connection from LA to ITCd	0.3–1
$\theta _{LB}$	Threshold for connection from LA to BA	0.1–0.6
$\theta _{BI}$	Threshold for connection from BA to ITCv	0.02–0.4
$\theta _{PI}$	Threshold for connection from PL to ITCd	0.01–1
$\theta _{II}$	Threshold for connection from IL to ITCv	0.1–1
$\varepsilon _{LI}$	Learning rate of connection from LA to ITCd	0.01–1
$\varepsilon _{LB}$	Learning rate of connection from LA to BA	0.01–1
$\varepsilon _{BI}$	Learning rate of connection from BA to ITCv	0.02–0.9
$\varepsilon _{PI}$	Learning rate of connection from PL to ITCd	0.01–1
$\varepsilon _{II}$	Learning rate of connection from IL to ITCv	0.01–1

#### Chamber choice

We evaluated CEA activity for each chamber exposure in test trials. We used the *softmax* equation on CEA activity values to find out the probability of choosing the conditioned chamber.

7 $$\begin{aligned} P(A) = \frac{e ^{q_{A}/T}}{\sum _i{e^{q_{i} / T}}} \end{aligned}$$

where *P*(*A*) is the probability of choosing the chamber A (CS1), $q_{A}$ is the activity of CEA when the animal looks at chamber A, $\sum {e ^{q_{i}}}$ is the sum of the exponential of CEA activity when the animal looks at chamber A and at chamber B, and T is a parameter (set to 0.7) called temperature, which regulates the steepness of the selection. In the simulations, a value of *P*(*A*) above 0.6 was considered to indicate a strong preference for chamber A, whereas a value within the range of (0.4, 0.6) was considered to indicate that the behavioural preference for the chamber had extinguished.

The probability of choosing chamber B was calculated as $1-P(A)$. To get a measure in seconds of the time spent in each chamber, we multiplied the probability value by 1200 since each behavioural test lasted 20 min. The central chamber was not simulated in the model.

#### Sensitivity analysis

To verify the robustness of the model and its predictions to parameter changes, we ran the following sensitivity analysis.

We launched our model with 98 million different combinations of parameters, using the Neuroscience Gateway Portal (Sivagnanam et al. 2013). We randomly sampled values for all parameters in Table 4 involving connection weights, plasticity thresholds and learning rates. The other parameters remained fixed for all neurons. Connection weights were uniformly drawn between 0 and 2 for fixed connections and between 0 and 0.2 when they referred to initial values of learned connections. Plasticity parameters were all uniformly drawn between 0 and 1. For each sample, we evaluated the corresponding behaviour on control conditions (no manipulation) and under the experimental manipulations listed in Table 3. Under each condition, we evaluated if the sample was able to acquire conditioning and then to extinguish it, and if its extinction happened sooner or later than controls.

Samples that replicated the results obtained in previous experiments (Table 3) were considered “valid”, while the others were discarded as implausible combinations of parameters since they did not fit available data. On control simulations, samples had to acquire a preference during the conditioning protocol and then extinguish it within the 14 days of extinction. We noticed that some samples would not learn anything until a few days into the extinction phase (i.e. on day 7), when NA inputs into mPFC was low enough. However, it is not plausible that the rats do not learn anything during the first few days or that their NA, without manipulations, is too high to enable learning. So we also enforced that, under control condition, samples had to start learning the extinction within the first 2 days. The day on which they completed the extinction was instead left free to vary within the 14 days available. All samples which satisfied these constraints under control and experimental conditions were then used to produce the result empirically tested here and to make predictions on the two new proposed experiments.

### Materials and methods of empirical experiment

#### Animals and drugs

A total of 13 male C57BL/6JIco (Charles River, Como, Italy) were purchased at 6–7 weeks of age and housed 4 per cage undergoing a 12-h light–dark cycle (lights on between 07.00 a.m. and 07.00 p.m.) for 3 weeks before experiments. All experiments were carried out in accordance with the Italian national law (DL 116/92 and DL 26/2014) on the use of animals for research based on the European Communities Council Directives (86/609/EEC and 2010/63/UE), and approved by the Italian Ministry of Health (license/approval ID $\#$: 10/2011-B and 42/2015-PR).

D-Amphetamine sulphate (Amph), chloral hydrate, 6-hydroxydopamine (6-OHDA) and GBR 12909 (GBR) were purchased from Sigma (Sigma-Aldrich, MI, Italy). Amph (2.5 mg/Kg) were dissolved in saline (0.9$\%$ NaCl) and injected intraperitoneally (i.p.) in a volume of 10 ml/kg. 6-OHDA was dissolved in saline containing Na-metabisulphite (0.1M).

#### Apparatus and behavioural paradigm

Behavioural experiments were performed using a CPP apparatus (Cabib et al. 2000). The apparatus comprised two grey Plexiglas chambers (15 $\times$ 15 $\times$ 20 cm) and a central alley (15 $\times$ 5 $\times$ 20 cm). Two sliding doors (4 $\times$ 4 cm) connected the alley to the chambers. In each chamber, two triangular parallelepipeds (5 $\times$ 5 $\times$ 20 cm) made of black Plexiglas and arranged in different patterns (always covering the same surface of the chamber) were used as conditioned stimuli.

The paradigm was performed as follows. On day 1 (pretest), mice were free to explore the entire apparatus for 20 min. During the following 8 days (conditioning phase), mice were confined daily for 40 min alternately in one of the two chambers. One of the patterns was consistently paired with drug (amphetamine, 2,5 mg/kg i.p.) and the other with vehicle (saline solution). This schedule lasted throughout conditioning.

Testing for the expression of CPP was conducted on day 10 using the pretest procedure. “EthoVision” (Noldus, The Netherlands), fully automated video tracking system (Spink et al. 2001), was used to collect and analyse behavioural data. Briefly, in this system, the experimental setup is recorded by a CCD video camera. The signal is then digitised (by a hardware device called “frame grabber”) and transmitted to the computer. EthoVision software was then used to analyse the digital data and obtain the “time spent” (in sec.), which was used as raw data for preference scores in each sector of the apparatus of each subject.

One week after the initial CPP test all animals were subjected to surgical procedures. Mice of the double depletion group were injected with GBR (15 mg/Kg) 30 min before 6-OHDA micro-injection to protect dopaminergic neurons. Five min after GBR injection all subjects were anaesthetized with chloral hydrate (450 mg/kg) and mounted in a stereotaxic frame (David Kopf Instruments, Tujunga, CA) equipped with a mouse adapter. Bilateral injection of 6-OHDA, 1.5 $\upmu$g/0.1 $\upmu$l/2 min or 1.5 $\upmu$g/0.08 $\upmu$l/2 min for each side were made, respectively, into PL (coordinates: + 2.8 AP; + 0.6 L; -1.3 V with respect to bregma) and into IL (coordinates: + 1.5 AP; + 0.3 L; -4.1 V with respect to bregma), through a stainless steel cannula (0.15 mm outer diameter, UNIMED, Swiss) connected to a 1 $\upmu$l-syringe by a polyethylene tube and driven by a CMA/100 pump. The cannula was left in place for an additional 2 min after the end of the infusion. Sham animals were subjected to the same treatment but received intracerebral vehicle. Coordinates and injection procedure were based on preliminary experiments.

One day after the surgery, all animals underwent a further test (re-test), to ascertain whether the surgery impaired the CPP. On the following day, the extinction procedure started. To investigate potential time-dependent differences in extinction, animals were exposed daily to CPP testing (20 min of non-confined extinction, Orsini et al. (2008). The extinction of the conditioned response was considered acquired after two consecutive days with no significant preference for the drug-paired chamber (Fricks-Gleason and Marshall 2008).
